# Adenosine, Energy Metabolism, and Sleep

**DOI:** 10.1100/tsw.2003.65

**Published:** 2003-08-20

**Authors:** Tarja Porkka-Heiskanen, Anna Kalinchuk, Lauri Alanko, Anna Urrila, Dag Stenberg

**Affiliations:** Institute of Biomedicine, University of Helsinki, Finland

**Keywords:** adenosine, caffeine, c-fos, energy metabolism, NF-kB, sleep, sleep deprivation

## Abstract

While the exact function of sleep remains unknown, it is evident that sleep was developed early in phylogenesis and represents an ancient and vital strategy for survival. Several pieces of evidence suggest that the function of sleep is associated with energy metabolism, saving of energy, and replenishment of energy stores. Prolonged wakefulness induces signs of energy depletion in the brain, while experimentally induced, local energy depletion induces increase in sleep, similarly as would a period of prolonged wakefulness. The key molecule in the induction of sleep appears to be adenosine, which induces sleep locally in the basal forebrain.

### WHY DO WE HAVE TO SLEEP?

Sleep is an essential part of the everyday life of animals as well as human beings. Despite intensive research, the key function(s) of sleep remain unknown. Indirect evidence suggests that whatever the function of sleep is, or whatever was the reason that sleep was developed in the course of evolution, it is vitally important. Three points in this respect are particularly relevant: (1) during the state of sleep the responsiveness of organisms to external stimuli is decreased, making them vulnerable to dangers of the surrounding world — still, practically all animals sleep, (2) sleep need is homeostatically regulated[1], which also suggests an important, life supporting function for sleep, (3) if rodents are kept awake for extreme time periods (several weeks) they die[2].

Sleep appears to have developed early in the course of evolution[3], suggesting that the core of sleep need is related to primitive functions of life, e.g., energy metabolism. Energy conservation, or the restoration of energy during sleep, has offered itself as an intuitive explanation of sleep propensity[4]. The replenishment of brain glycogen stores has also been suggested as a possible cause of sleep[5].

## BRAIN ENERGY METABOLISM

### Sources of Brain Energy

According to the classical view, oxidation of glucose (Fig. 1) is the main source of energy in the brain, while lactate, produced by anaerobic glycolysis, is used only in pathological conditions[6]. More recent studies have revealed that neurons use lactate as their energy source also under normoxic conditions, and may even prefer it over glucose during neuronal activation[7,8]. Glucose is metabolized to lactate in astrocytes and transferred to neurons[7,8], where lactate dehydrogenase converts it to pyruvate, which is further oxidized to produce ATP. In addition to blood glucose, the brain has a small energy reservoir in the form of glycogen, that is stored in astrocytes. The total glycogen content in astrocytes is small: as a sole source of energy it could keep up brain activity for less than 5 min[9].

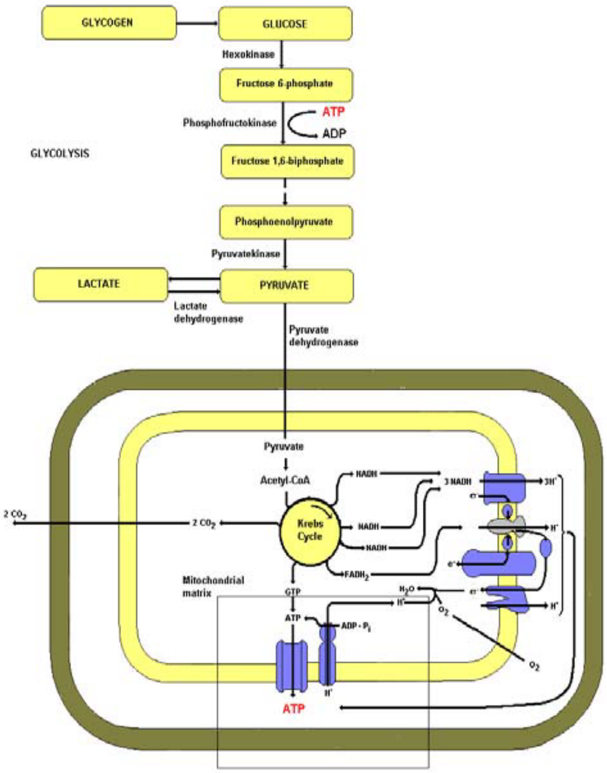

**FIGURE 1.** Main phases of energy metabolism. Glycolysis: glucose is converted to pyruvate. Pyruvate can be metabolized either to lactate to produce 2ATP, or enter the citric acid cycle to produce 36 ATP.

### How is Brain Energy Used?

The main part of brain energy is used to sustain neuronal activity: it has been estimated that in the human brain, glutamate/glutamine cycle is the major metabolic flux with a rate of about 80% of glucose oxidation[10]. There is thus a close relation between the activity level of neurons and energy consumption in the brain (for review see [7]).

Stimulation of the brain (sensory or cognitive) will increase lactate in the stimulated area[11,12,13]. The elevation in lactate coincides with the growing energy demand of increased neuronal activity[14]. The origin and exact role of lactate in the course of stimulation are not clear. According to Magistretti et al., increased neuronal firing would stimulate the use of lactate as a neuronal fuel, leading to elevated lactate level[7]. Another possible mechanism of lactate elevation would be the glycogen shunt: glucose is rapidly metabolized from glycogen in astrocytes[8]. This view is supported by the finding that tactile stimulation in rats decreased glycogen levels, while lactate levels were elevated[15].

### ENERGY METABOLISM AND SLEEP

#### General

During non-REM sleep, brain energy expenditure decreases; cerebral blood flow[16] and the cerebral metabolic rate decrease[17,18], while glucose[19,20] and ATP concentrations increase[19,21]. Decreased extracellular lactate and pyruvate concentrations during sleep further indicate a reduction in energy metabolism[19,20,21].

During prolonged wakefulness, energy is utilized continuously and brain extracellular glucose concentrations[20], as well as phosphocreatinine and ATP levels, decrease[19]. At the cellular level, waking activates mitochondrial genes related to energy production (e.g., subunits of cytochrome c oxidase, NADH dehydrogenase, and 12S rRNA)[22]. PET studies in humans have shown that the cerebral metabolic rate for glucose is decreased after prolonged wakefulness[23,24].

#### Energy Depletion and Sleep

The relationship between energy depletion and sleep induction has been addressed experimentally: the *intraperitoneal* infusion of 2-deoxy-D-glucose, a glucose antimetabolite, increased sleep dose dependently in cats[25], while in diabetic patients experimentally induced mild hypoglycemia for 1 h during the night generated subjective feelings of fatigue during the following day[26]. We used 2,4-dinitrophenol (DNP), a molecule that prevents the synthesis of ATP, to induce local energy depletion in the basal forebrain. DNP infusions (3-h) induced elevations in extracellular concentrations of lactate, pyruvate, and adenosine, as well as increases in non-REM sleep during the following night. Sleep was not affected when DNP was administered to adjacent brain areas, although the metabolic changes were similar. The amount and the timing of the increase in non-REM sleep, as well as in concentrations of lactate, pyruvate, and adenosine with 1.0 mM DNP infusion, were comparable to those induced by 3 h of sleep deprivation[27]. The use-dependent regulation of sleep propensity, possibly involving stimulus-activated changes in energy metabolism, has been suggested by observations that local brain stimulation (e.g., tactile stimulation on of the hand to stimulate the respective area in the motor cortex) during waking evokes changes in subsequent sleep[28,29]. In humans, the normal metabolic response to stimulation (increase in extracellular lactate) disappeared in the course of prolonged wakefulness[30]. The rapid and temporary increase in lactate on stimulation represents the brain's metabolic response to rapid energy demand with increased neuronal activity. It is thus conceivable that while the brain's energy resources become restricted during prolonged wakefulness, the demand of rapid production of energy during neuronal activation will not be met. The behavioral effects of prolonged wakefulness may arise from dysfunction of this energy production mechanism.

## Adenosine

### General

Adenosine (AD) is a ubiquitous molecule, directly associated with energy metabolism (see Fig. 2), but also functions in cellular communication and regulation of neural activity. Pharmacological studies have shown that adenosine and its agonists have sleep-inducing effects[31,32]. Furthermore, the well-known stimulants caffeine and theophylline have been determined to be antagonists of AD receptors[33]. Acting on the widely distributed A1 receptor, AD is generally an inhibitory neuromodulator reducing the activity of neurons in a number of brain regions, either by directly hyperpolarizing the postsynaptic membrane, or by acting presynaptically to decrease the release of excitatory neurotransmitters (for review see [33,34,35]). However, AD may also have an indirect excitatory effect on postsynaptic neurons, via A1-mediated presynaptic inhibition of inhibitory neurotransmitter release. Although most of the physiological effects of AD in the brain are mediated by the A1 receptor, AD action at the A2 receptor can have neuromodulatory effects that oppose action at A1 receptors (reviewed in [33,34]).

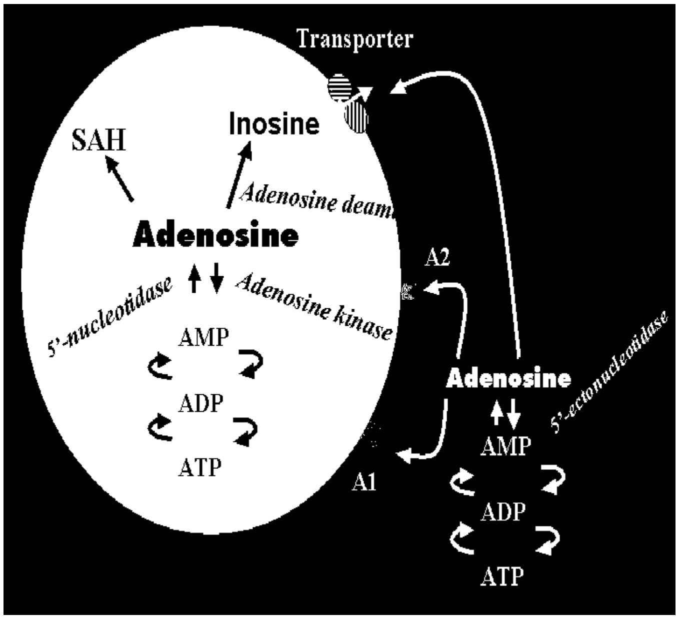

**FIGURE 2**. Schematic of main intra- and extracellular metabolic pathways of adenosine. Adenosine is formed from AMP intracellularly by the enzymes ATP-ase, ADP-ase, and 5'-nucleotidase, and extracellularly by the respective ectoenzymes. Adenosine kinase converts adenosine to AMP, while adenosine deaminase converts it to inosine. The third enzyme to metabolize adenosine is S-adenosylhomocysteine hydrolase, which converts adenosine to S-adenosylhomocysteine (SAH). Adenosine concentration between the intra- and extracellular spaces is equilibrated by nucleoside transporters. Adenosine's effects on cells are mediated through specific receptors. Abbreviations: A1, A1 adenosine receptor subtype; A2, A2 adenosine receptor subtype; ADP, adenosine diphosphate; AMP, adenosine 5'-monophosphate: ATP, adenosine triphosphate (modified from [36]).

### Adenosine and Natural Sleep

Using *in vivo* microdialysis in cats we collected samples for adenosine analysis during spontaneous sleep-waking cycle. Adenosine levels in samples collected during waking were higher than during periods of slow wave sleep (SWS) in all brain areas. Adenosine levels during sleep were approximately 75 to 80% of waking values, and similarly reduced in all brain regions[36]. Thus, adenosine levels vary with behavioral state, the levels being higher during wakefulness than sleep.

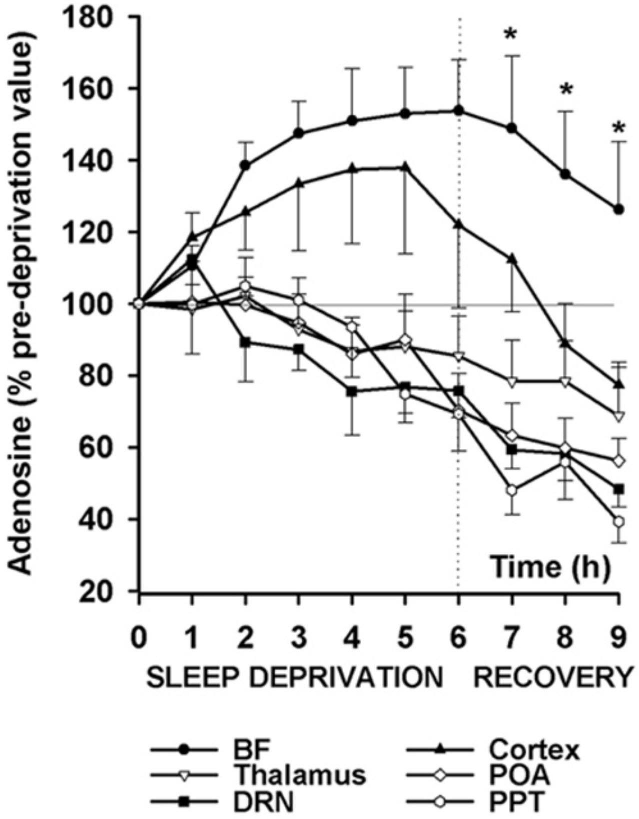

**FIGURE 3**. Adenosine concentrations in different brain areas during sleep deprivation and recovery sleep. Cats were kept awake for 6 h. Prior to the beginning of the sleep deprivation, samples were collected to obtain baseline wakefulness values for each probe (predeprivation value = hour 0; defined as 100%). Two patterns were evident in the sleep deprivation–induced changes in adenosine; in the basal forebrain (BF) and cortex adenosine levels could be seen to rise during the sleep deprivation, whereas in the other four areas adenosine levels were either stable or declined slowly during the 6 h of sleep deprivation. During recovery, sleep adenosine concentrations were significantly higher in the BF than in all other areas. Significant differences in adenosine concentration between BF and all other areas are marked with asterisks. Abbreviations: DRN, dorsal raphe nucleus; POA, preoptic hypothalamic area; PPT, pedunculopontine tegmental area (modified from [36].

#### *Adenosine and Prolonged Wakefulness*

Measured using *in vivo* microdialysis as described above, adenosine concentrations increased in the basal forebrain steadily in the course of 6-h sleep deprivation, reaching at 6 h twice the values that were measured in the beginning of the sleep deprivation period[36,37]. During the recovery sleep period, adenosine levels in the basal forebrain gradually decreased over the next 2 to 3 h, a time when the behavioral state was mainly SWS. However, when samples were collected in different brain regions during 6-h total sleep deprivation, we found quite different patterns. The rise in adenosine levels in the cortex site paralleled those in the basal forebrain (BF) during the first 5 h, although the rise was not as steep as in the basal forebrain, and started to decline in the last hour of deprivation. In the VA/VL (thalamus), dorsal raphe nucleus, pedunculopontine tegmental nucleus, and preoptic area adenosine levels did not rise during 6-h sleep deprivation, though many of these regions are also known to be active in sleep regulation. Interesting regional differences in extracellular adenosine concentrations were also found during the recovery sleep that followed sleep deprivation. During the 3-h recovery sleep period, adenosine concentrations in the basal forebrain declined slowly, remaining significantly higher than the predeprivation basal wakefulness level at 3 h of recovery sleep (Fig. 3). In all other brain areas, the adenosine concentrations were either at the level of predeprivation values, or lower throughout the recovery sleep period. We have repeated the key experiments in rats, and shown that extracellular adenosine concentrations also in this species are lower during sleep, and that adenosine accumulates in the basal forebrain of rats during sleep deprivation[38].

These data indicate that during prolonged wakefulness, extracellular adenosine accumulates selectively in the basal forebrain and, also, to a lesser extent, in the cortex. This unique pattern of regionally specific rises in adenosine in response to sleep deprivation supports the hypothesis that adenosine promotes the transition from wakefulness to SWS by inhibiting the discharge activity of wake-active cortically projecting neurons of the basal forebrain, and possibly also by inhibiting neurotransmitter release from the cortical terminals of these basal forebrain neurons.

### *Effects of Chemically Induced High Brain Extracellular Adenosine Levels on Sleep*

NBTI, an inhibitor of nucleoside ENT1 type transporter, was used to locally increase extracellular adenosine concentration in the brain. When NBTI was perfused unilaterally to the test areas (basal forebrain and thalamus), the extracellular adenosine concentration doubled in both areas, reaching the same level that was measured after the 6-h sleep deprivation in the basal forebrain. While the increase in extracellular adenosine concentration was similar in both the cholinergic basal forebrain area and in the thalamus, the effects of the increased adenosine concentration on vigilance state were dramatically different. When perfused to the basal forebrain, NBTI decreased waking and increased both SWS and REM sleep, while when perfused to the thalamic VA/VL nucleus, the vigilance states remained unchanged[37]. These data indicate that not only the increase in extracellular adenosine concentrations but also the sleep-inducing effect of adenosine is specific to the basal forebrain.

### *Long-Term Effects of High Adenosine Levels*

While many of the somnogenic effects of adenosine may represent direct receptor-mediated actions, sleep deprivation has long-duration effects with a time course that suggests that they are likely to be mediated through effects on transcription. For example, in humans successive nights of sleep restriction have a cumulative effect on neurobehavioral alertness[39] and the effects of prolonged sleep deprivation in animals are not recovered during a single night of recovery sleep[40]. The extracellular levels of adenosine start to decrease as soon as sleep is initiated. The extension of the effects of prolonged wakefulness over several days suggests that intracellular events, including protein synthesis, may be activated. We have shown that adenosine administration increases A1 receptor mRNA[41] and the DNA-binding activity of the transcription factor NF-kB in response to prolonged wakefulness[42] in the basal forebrain, implying initiation of an A1 receptor–mediated protein synthesis in this area.

### BASAL FOREBRAIN AND SLEEP

Several experiments suggest that the basal forebrain has a specific role in adenosine-mediated sleep induction. The elevation of adenosine, lactate, and pyruvate during prolonged wakefulness occurs only in the basal forebrain[27,36]. Also the sleep-inducing effects of NBTI-induced elevation of adenosine level and experimentally induced energy depletion are restricted to the basal forebrain[37]. Increases in A1 receptor mRNA[41] and in the DNA-binding activity of the transcription factor NF-kB in response to prolonged wakefulness[42] are also specific to the basal forebrain. Moreover, A1 receptors activate a Ca2+-mediated intracellular signal transduction pathway specifically in the cholinergic cells[43], offering a potential mechanism that could explain the site-specific effects of adenosine. Taken together, these data strongly suggest a specific role for the basal forebrain in the adenosine-mediated regulation of vigilance states. As experimentally induced energy depletion elevated the extracellular concentrations of adenosine, lactate, and pyruvate in all brain areas, while sleep deprivation did so only in the basal forebrain, it may be argued that prolonged wakefulness generates energy depletion more easily in the basal forebrain than in other brain areas. The wakefulness-promoting cells in the cholinergic basal forebrain could therefore act as energy level sensors, or as fuses to protect the rest of the brain from the injurious effects of energy depletion.

One-third of the cortically projecting magnocellular basal forebrain neurons are cholinergic, one-third GABA-ergic, while one-third are unidentified[44]. The cholinergic cortical and thalamic projections are thought to be essential for the induction of cortical EEG arousal[45]. We have previously suggested that these projections could be central to the effects of adenosine on vigilance states[37]. Adenosine inhibits virtually all neurons via A1 receptors, but the inhibitory action is more prominent on excitatory cells, so that the net effect in most brain areas is a reduction in neural activity[46]. To induce a decrease in the cellular activity of cortical neurons, adenosine should act on the excitatory neurons in the basal forebrain. *In vivo* measurements in freely behaving animals show that adenosine inhibits the waking-active neurons in the basal forebrain[47]. As cholinergic neurons are effectively inhibited by adenosine[48], they appear to be the most obvious targets for adenosine. However, it is possible that the modulation of the activity of other projection neurons could also be of importance in mediation of the sleep-inducing effects of adenosine.

### CONCLUSIONS

The continuous activity of the wakefulness-promoting cells in the basal forebrain imposes a burden on the mitochondrial energy production capacity during wakefulness. When wakefulness is prolonged, these cells start to suffer from an energy deficit, resulting in increased glycolysis and elevations in extracellular concentrations of lactate, pyruvate, and adenosine. The enhanced extracellular concentration of adenosine contributes to the increased sleep propensity and promotes the transition from wakefulness to sleep. Adenosine may affect sleep through several mechanisms; the immediate effect, the decrease in neural activity, may promote the transition from wakefulness to sleep[37,48], while intracellular events, mediated through A1 receptors, may be of importance in the induction of sleep propensity[41,42].

The increase in lactate on stimulation represents the brain's metabolic response to rapid energy demand with increased neuronal activity. It is thus conceivable that while the brain's energy resources become restricted during prolonged wakefulness, the demand of rapid production of energy during neuronal activation will not be met. The symptoms of prolonged wakefulness could arise from malfunctioning or absence of this mechanism: neurons could still function, but more slowly and less effectively.

### ACKNOWLEDGEMENTS

We thank Dr. Radhika Basheer, Dr. Robert E. Strecker, and Dr. Robert W. McCarley at Harvard Medical School for their valuable input to the experiments described above. The research was funded by The Academy of Finland, Finska Läkaresällskapet, EU grant QLK6- CT-2000-00499, CIMO and the Juselius Foundation.
